# A review of youth mental health in the U.S.: the current landscape, youth perspectives, and strategies for equitable solutions

**DOI:** 10.3389/frhs.2026.1837334

**Published:** 2026-05-20

**Authors:** Tasha L. Golden, T. W. Cherry Ng, Aanchal Mohanty, Suzanne J. Block, Alyssa Tiedemann, Susan Magsamen

**Affiliations:** 1 International Arts and Mind Lab, Johns Hopkins University School of Medicine, Baltimore, MD, United States; 2 Department of Health, Behavior and Society, Johns Hopkins Bloomberg School of Public Health, Baltimore, MD, United States

**Keywords:** health equity, mental health resources, youth engagement, youth mental health, youth perspectives

## Abstract

The youth mental health crisis in the United States has garnered increasing attention, stimulating calls for urgent, evidence-based action. To inform the development of effective solutions, this literature review synthesized current data on youth mental health challenges, youth perspectives on needs and priorities, and existing resources and gaps. Findings reveal significant increases in mental health concerns among youth; disparities in outcomes and access to care, particularly among marginalized populations; and the critical importance of youth engagement and leadership in co-developing solutions. Youth perspectives emphasized the need for mental health education, school- and community-based resources, and arts-based activities as key avenues for fostering social connection and engagement. The review describes three categories of existing resources (community-based services, national nonprofits, and government initiatives), notes the potential value of creative engagement, and identifies gaps and challenges—such as inadequate funding and barriers to access. Findings underscore the urgent need for equity-oriented, culturally responsive approaches, including modalities informed by the arts, to address the youth mental health crisis. Importantly, they indicate the value of co-learning and co-strategizing with youth to promote mental health and flourishing. Despite some limitations, this review offers a timely synthesis of key themes and priorities, providing actionable insights for intervention and research efforts that support the mental health and well-being of youth in the United States.

## 1 Introduction

The mental health of youth in the United States has emerged as a significant focal point for community and national health initiatives. In 2021, the U.S. Surgeon General, Dr. Vivek Murthy, underscored the urgency of this issue in his advisory titled, *Protecting Youth Mental Health*, which states that “the challenges today's generation of young people face are unprecedented and uniquely hard to navigate.” Subsequently, in May 2023, the Biden-Harris administration announced measures aimed at confronting what it termed the “nation's mental health crisis,” dedicating particular attention to youth mental health. This was further reinforced in July 2023 by the National Governors' Association's release of “Strengthening Youth Mental Health: A Governor's Playbook.” In a 2023 national survey of youth in the U.S., 20.3% of youth had a current diagnosis of a mental or behavioral health condition, and mental health challenges among youth appear to have been exacerbated by the COVID-19 pandemic (1, 2). These statements and documents signal a unified call to action to better protect and support youth mental health.

The development of effective action steps for addressing youth mental health requires up-to-date knowledge not only of current mental health statistics and concerns, but also of youth perspectives, community and cultural factors, health disparities, and nonclinical interventions that together illuminate needs, priorities, barriers, and opportunities. Evidence indicates that the effectiveness of youth mental health interventions is affected by youths' engagement in intervention development (3–5). Previous studies have also indicated the profound mental health impacts of social drivers and community factors (6, 7), arts and cultural engagement (6, 8) and social connection (9–11). In addition, disparities in mental health outcomes point to the value of population-specific, culturally-responsive approaches (12, 13). The current literature review was therefore undertaken to provide an updated knowledge set that considers each of these essential factors, with the aim of supporting the development of strategies, interventions, policies, and other solutions to bolster youth mental health.

## 2 Methods

### 2.1 Search strategy

This narrative literature review aimed to gather and convey data regarding current youth mental health challenges in the US, youths' explicitly-named needs and requests related to mental health, health disparities across diverse youth populations, community and cultural factors and solutions, current evidence-based programs, and reported gaps in services.

To this end, electronic databases were searched for articles relating to the above, as well as to youth voice, participatory research, and community-based, nonclinical interventions such as arts and culture engagement. Beginning with broad searches and iteratively narrowing based on findings, the research team conducted multiple layered searches of youth mental health studies and youth-led perspectives; for example, the team actively sought reports indicating what youth themselves perceive as important and relevant for their mental health, and the kinds of interventions and changes they request.

Using Web of Science, APA PsychInfo, and PubMed, searches were also conducted to identify scales, questionnaires, and surveys that focused on youth mental health or on arts/culture interventions among U.S. youth between 2018 and 2023. A total of 37 such measures were captured and analyzed by the research team, 20 of which were deemed “mental health forward” and 17 of which were “arts and culture forward.” A sample of these measures can be found in Table 1.

**Table 1:** Study measures.

Type of measure	Name of measure
Mental Health-forward Measures in Studies	Patient-Reported Outcomes Measurement Information System (PROMIS) anxiety scale
	Greater Engagement in Gender-Sexuality Alliances (GSAs)
	GSA Characteristics Survey
	Brief Problem Monitor
	Youth Self Report
	COVID-19 Experiences Survey
	Adolescent Behaviors and Experiences Survey (ABES) (adapted version for a study)
	Adolescent Behaviors and Experiences Survey (ABES) (original)
	Teen and Parent Surveys of Health (TAPS)
	The Trevor Project: 2022 Poll on Negative Impacts of Anti-LGBTQ Policies on LGBTQ Youth
	National Alliance on Mental Illness (NAMI) Teen Survey
	Strengths and Difficulties Questionnaire
	Youth Risk Behavior Survey
	The Traumatic Events Screening Inventory for Children (TESI-C)
	The Patient Health Questionnaire-9
	Minnesota Student Survey
	Patient Health Questionnaire-2
	Greater Engagement in Gender-Sexuality Alliances (GSAs) and GSA Characteristics Survey
	Patient-Reported Outcomes Measurement Information System (PROMIS) anxiety scale
	Interpersonal Needs Questionnaire
	The Depression Anxiety Stress Scales 21 (DASS-21)
	General Self-Efficacy Scale (GSES)
Arts and Culture-forward Measures in Studies	Arts and Cultural Engagement, Reportedly Antisocial or Criminalized Behaviors, and Potential Mediators Questionnaire
	Early Childhood Longitudinal Study (ECLS)
	The National Longitudinal Study of Adolescent to Adult Health
	National Education Longitudinal Study of 1988
	The Early Childhood Longitudinal Study (ECLS)
	2016 National Survey of Children’s Health
	The Child Behavior Checklist
	The Sports and Activities Questionnaire
	Center for Epidemiologic Studies Short Depression Scale
	Youth Experiences Survey (YES)
	Music Participation Scale
	Positive Youth Development Questionnaire
	The out-of-school time (OST) structured activity
	Hemingway Measure of Adolescent Connectedness
	Hopeful Future Expectations Scale
	Kidscreen-27
	The Taking Party Survey
	Middle Years Development Instrument

Once initial findings were written up, key scholars in youth development were invited to review the work, provide feedback, and recommend additional studies. The scholars were identified through the research team's existing professional networks, prioritizing individuals with established contributions to youth development research, practice, and/or policy. These individuals were selected based on their subject-matter expertise and familiarity with the field.

Scholars were recruited via direct email outreach and invited to review the first draft of the review. All feedback and additional works were then analyzed and incorporated via discussion and iterative revisions by the research team.

### 2.2 Inclusion criteria

This search included articles and reports that were accessible in English, published between 2000 and 2024, inclusive of information regarding youth mental health in the U.S. (per the aims of the search), and published in peer-reviewed journals and/or by credible government or other nonprofit entities. Given the exploratory nature of the search, no formal exclusion criteria were applied.

### 2.3 Analysis

Included articles in this narrative literature review were analyzed in terms of aim, measures, and key findings. Relevant findings were ultimately compiled and categorized into three broad themes: State of Youth Mental Health, Youth Perspectives, and Existing Resources. In each category, subthemes were iteratively developed by the research team and key reviewers through multiple rounds of discussion, during which thematic boundaries were refined and resolved through consensus; final themes and subthemes are represented in Table 2.

**Table 2:** Literature review themes and subthemes

Theme	Subthemes
State of youth mental health	Overview
	Health Disparities
	Access to Care
	Disparities in Access
	Effects of the COVID-19 Pandemic
	Neurobiological Factors
	Additional Factors Affecting Mental Health
	Impacts of Youth Mental Health Challenges
Youth perspectives	“Nothing About Us Without Us”—Youth Leadership
	What Youth Have Requested
Existing resources	Community-Based Resources
	National Nonprofit Resources
	Government Resources

## 3 Results

### 3.1 The state of youth mental health

#### 3.1.1 Overview

Mental health challenges among U.S. youth have been the leading cause of disability and adverse life outcomes for many years, with up to one in five children ages 3–17 reporting a mental, emotional, developmental, or behavioral disorder (14). Alarmingly, nearly half of the 7.7 million children with treatable mental health disorders fail to receive adequate treatment (15). Over the past decade, data have shown a striking surge in the prevalence of anxiety, depression, and other behavioral challenges (16); from 2016 to 2020, the number of youth aged 3–17 years diagnosed with anxiety and depression increased by 29% and 27%, respectively. In 2021, the Youth Risk Behavior Survey indicated disturbing rates of suicidal tendencies among high school students, with approximately 23.6% of female students and 11.6% of male students reporting having made a plan, 30% of female students and 14.3% of male students reporting having seriously considered attempting suicide in the last year, and 13.3% of female students and 6.6% of male students having attempted suicide in the last year (17). Correspondingly, data from 2011 to 2020 indicate that psychiatric visits to emergency departments for mental health challenges increased from 4.8 million to 7.5 million (18). While the total number of youth has also increased over time, these percentages indicate an increasing proportion of youth experiencing significant mental health challenges (19).

#### 3.1.2 Health disparities

In general, greater mental health risks are experienced by youth from minority ethnic, religious, or other groups facing exclusion or prejudice; youth who are neurodivergent or have learning disabilities; youth exposed to stigma; and youth lacking equitable access to quality healthcare (20). Socioeconomically disadvantaged youth are two to three times more likely to develop mental health conditions than their peers with higher socioeconomic status (21). Moreover, youth facing unstable housing or homelessness are also at a heightened risk for adverse mental health outcomes, with up to 33% of youth experiencing homelessness reporting attempted suicides, compared to 2% of stably housed youth (22). Youth who experienced homelessness also had a higher incidence of self-injury, suicidal ideation, and depressive symptoms (22). Belonging to multiple marginalized groups also compounds risk to mental health and wellbeing (23). Notably, persistent structural and racial inequalities, including first and second-hand experiences of racism such as the deaths of Black Americans at the hands of police officers and COVID-related violence against Asian Americans, have contributed to psychological distress that impacts youths' mental health and wellbeing (24, 25). Given ongoing inequalities and the extent of youth mental health concerns, efforts to improve youth mental health require an equity-oriented, culturally-responsive lens.

#### 3.1.3 Access to care

The literature reveals that youth often face barriers to access to mental health treatment, including primary and secondary care, evidence-based treatment, and engagement with the mental health infrastructure (12, 26–30). Although the Affordable Care Act (ACA) has increased rates of youth access to care across all racial and ethnic backgrounds (31), fewer than half of U.S. adolescents have access to quality mental health care. In fact, the Department of Health and Human Services reports that 11.4% of youth in the United States suffer from a serious mental illness (SMI), yet only 57.9% of these youth are able to access treatment (32). Barriers to care exist at multiple intersecting levels of social-ecological impact, including the individual (e.g., navigation, socioeconomic status, knowledge of resources, negative care experiences), interpersonal (e.g., lack of youth-friendly services; cultural/linguistic barriers), organizational (e.g., staffing, stringent consent laws), community (e.g., stigma, transportation), and public policy levels (e.g., financial impact) (33). Notably, there is an estimated one youth psychiatrist for every 10,000 youths, and location factors into access as well—as nearly 80% of rural counties lack a single psychiatrist (34, 35).

#### 3.1.4 Disparities in access

Disparities in access to mental health care among youth have been reported along the lines of socioeconomic status, race and ethnicity, gender identity, sexuality, location, and systems involvement (36). For example, in comparison within youth populations, youth living in poverty are the least likely among the general youth population to be connected with high-quality mental health care, with less than 15% of children experiencing poverty receiving services (37). This is likely due to a range of barriers, including lack of insurance, clinic hours that do not accommodate inflexible work schedules, and ongoing gaps in accessible mental health services under managed care plans (37, 38). Moreover, minors living in states such as Arizona, Delaware, and North Dakota must obtain parental consent to access inpatient and outpatient mental health treatment—a legal factor that may decrease youth's decision-making autonomy and access to treatment choices that best resonate with the individual (39).

Importantly, youth of color face disproportionate barriers in accessing mental health care due to structural, social, and economic inequities such as lack of health insurance coverage and financial and logistical barriers to accessing care (40, 41). In addition, the number of clinicians of color is not representative of the population; as a result, youth of color have less access to clinicians with cultural knowledge or expertise, and they often face bias and racism from providers (42). The lack of a diverse and culturally competent mental health care workforce, and the absence of culturally informed treatment options, were found to be barriers to seeking and obtaining mental health care (43). Gender expansive and LGBTQIA youth also face challenges in care access due to a lack of appropriately trained healthcare providers who are experienced working with this population (44). Lastly, approximately 70% of youth in the juvenile justice system have a diagnosable mental health disorder (45), yet as few as 20% of them receive mental health services after their first contact with the juvenile court system (46). Because justice-impacted youth are disproportionately members of racially/ethnically marginalized groups, and live at or below the poverty line (47, 48), these systems-based barriers exacerbate broader health inequities.

#### 3.1.5 Effects of the COVID-19 pandemic

The rising rates of psychological distress among youth were exacerbated by pandemic-related stressors such as disrupted routines and relationships, COVID-19 infections, and deaths of loved ones. For example, due to the pandemic's high death toll, many youth lost close relations during the pandemic—an experience whose difficulty was compounded by lack of access to collective structures for grieving, such as funerals and family gatherings (49). Many young people were forced to bid goodbye to family and friends through digital means (50). The onset of grief due to loss in the pandemic appears to have created secondary problems such as anxiety, depression, and substance abuse (50).

Beyond grief, public health measures during the pandemic often created or exacerbated social isolation and loneliness, which have been associated with increased anxiety, depression, and suicidal ideation among youth (51). Recognizing signs of child abuse and mental health concerns also became more difficult as pandemic-related measures reduced in-person interactions, limiting opportunities to notice or disclose experiences of maltreatment (52).

When comparing data from pandemic and pre-pandemic periods, studies report longitudinal deterioration in the mental health of youth, including increased depression and anxiety (53–55)—indicating that the pandemic may have accelerated long-term upward trends in the prevalence of mental health challenges faced by youth. For example, among a group of females who reported seriously considering suicide, the prevalence of suicidal thoughts and behaviours increased from 24% to 30% from 2019 to 2021 (17)—continuing a pattern of increasing rates of mental health concerns prior to the pandemic. Importantly, given that COVID-19 has disproportionately impacted communities of color and low socioeconomic status (56–58), the considerable mental health impacts of illness, grief, and isolation have also disproportionately affected these communities (59)—thus exacerbating ongoing mental health inequities.

#### 3.1.6 Neurobiological development

Youth mental health is also affected by neurobiological development. Neuroplasticity, the process by which nerve cells modify and change connections in response to stimulation and experience (60), is critical for learning, memory, and recovery as the brain adapts to the environment. During adolescence, the brain undergoes peak synaptic pruning, after which it loses plasticity in some areas (61). During this time of critical change, the brain is particularly sensitive to environmental experiences—which can have differential effects on social, psychological, and neural processes based on the child's developmental stage (62, 63).

Due to the significance of this period of development, early adversity has been linked to academic, social, and mental health problems later on (64), as have early substance use and addiction during adolescence (64). These experiences also appear to be predictive of depression and the emergence of psychopathologies in adulthood (65, 66), potentially through mediators of plasticity-related molecular processes such as brain-derived neurotrophic factor (BDNF) (67). More broadly, studies indicate that adverse childhood experiences (ACEs) such as poverty, child abuse, and the death of a family member are associated with longterm mental health concerns (68). Related, positive childhood experiences (PCEs) have been shown to counter effects of negative experiences, due in part to the high-level of brain plasticity observed during this developmental period (69, 70). PCEs refer to factors that increase the likelihood of improved youth development, resiliency, and adaptive skills, such as feeling supported by family and friends, enjoying participation in community traditions, and feeling safe and protected by an adult in the home (71). Other protective factors include having access to education, housing, and food; an ability to regulate emotions and attend to relevant stimuli; the ability to make adaptive decisions; and healthy parenting (70, 72).

#### 3.1.7 Additional factors affecting mental health

Beyond the factors noted above, upward trends in the reporting of mental health challenges may be traceable in some part to youths' increasing willingness to openly discuss such challenges (73). However, research also makes it clear that young people's mental health has been affected by sociopolitical challenges such as the national reckoning over the deaths of Black Americans at the hands of police officers (74); growing concerns about natural disasters and climate change (75); rising income inequality (76); racism (25); gun violence and mass shootings (77); pressures faced at school (78); and increasingly polarized political dialogue (79).

It is important to note that, while much has been made of correlations between upward trends in mental health challenges and rises in the use of digital technology and social media, recent research challenges the conflation of correlation with causation (80, 81). A 2023 consensus report from the National Academies reviewed the literature, finding insignificant associations between daily use of social media and adolescent wellbeing, though it noted some harms as well as benefits that have emerged from research, and called for further study (82). Given the pervasive role of digital media in youth development, and the emerging roles and applications of artificial intelligence, further investigation is warranted to better understand its effects on youth mental health.

It is clear that mental health is top of mind for young people. The literature makes clear that mental health is a top concern among youth people. A 2024 survey of 12–17-year-olds by Common Sense Media found that the participants' greatest concern related to young people's health and wellbeing was indeed mental health challenges (30%), which outweighed gun violence (21%), social media (20%), and addiction and drug use (19%) (83).

#### 3.1.8 Impacts of youth mental health challenges

The impacts of mental health challenges are experienced across multiple domains of life, causing decreased academic achievement, strained familial and peer relationships, and poorer physical wellbeing (84). Youth with mental health challenges are also at greater risk for substance use, experiencing violence, and sexual behaviors that put them at an elevated risk for HIV, unintended pregnancy, and other sexually transmitted diseases (84). In addition to the many direct individual effects of mental illness, there are significant economic impacts. The global cost of youth mental health conditions is estimated to be $16.1 trillion between 2010 and 2030, a number that is likely to have increased as a result of inflation and the impact of COVID-19 on youth mental wellbeing (85).

Notably, beyond the negative ripple effects of mental illness, it is important to consider the simple reality that mental health challenges often prevent young people from leading the full, flourishing lives they seek. The World Health Organization's (WHO) definition of health includes not only the absence of disease but also a state of “complete…wellbeing” (86). It is therefore critical to consider not only how to reduce and prevent the negative effects of mental illness, but how to support youth to thrive and flourish within an integrated whole-person and whole-system framework by increasing access to opportunities associated with positive experiences of gratitude, optimism, self-esteem, and happiness (87), as well as creativity (88), self-compassion (89), and satisfaction with life (90). For example, a recent study found that self-regulation skills such as mindfulness and self-compassion appeared to provide buffers against COVID-related anxiety amongst youth (91). Building a healthy future requires creating structures and cultures in which youth not only have access to quality reactive care, but equitable, proactive opportunities to develop and maintain a state of flourishing.

### 3.2 Youth perspectives

Given the needs outlined above, the following section examines the role of youth engagement and leadership, and describes some of the mental health supports that youth have requested.

#### 3.2.1 “Nothing about US without US”—youth leadership

The literature indicates that it is crucial to elicit, value, and follow young people's leadership to collaboratively design and implement mental health interventions and activities. Youth interventions are best led by youth themselves, and youth have a developmental need to be in environments that promote their autonomy. The Disability Rights community has elevated the mandate, “Nothing about us without us” (92), indicating that decisions and services should be led by those most affected by them. This mandate applies to any work designed to address equity and care among vulnerable and/or minoritized populations, such as youth.

Findings point to recommendations for fully integrated youth partnerships that move beyond one-time participation in a given health care service to involve youth in service planning, implementation, and monitoring (93, 94). This engagement is not only equitable and participant-centered, but also more effective. Research has also indicated that youth-oriented solutions that are created *without* youth perspectives are likely to fail at addressing health disparities, barriers in access to care, and issues related to access and health literacy. By contrast, multiple studies have indicated that youth engagement and autonomy-supportive practices promote patient-centered care (95–97). Moreover, youths' insider knowledge on issues that impact them and their communities can help them identify developmentally appropriate and culturally relevant questions, problems, and solutions that traditional research may miss (98). For example, youth participatory action research (YPAR) engages youth in advocating for changes that they value through an iterative cycle of research, action, and evaluation (98). Youth have engaged in YPAR using methods such as surveys, interviews, observations, photovoice, and mapping to address a wide range of health equity issues such as unequal access to healthy food and physical activity opportunities, lack of safe and accessible outdoor open spaces, and exposure to hormone-disrupting chemicals in pesticides and personal care products (99–102).

Some examples of current youth mental health supports that are led “by youth, for youth” include: Mental Health Kingdom, a virtual mental health community led by certified youth and young adult peer support specialists via Discord; Mindful Minute, a student-led organization that teaches mindfulness skills and the integration of mind-body practices into the school day; and DMV Students for Mental Health Reform, which advocates for policies such as excused absences for mental health days and the reallocation funds towards increased student support staff (103).

#### 3.2.2 What youth have requested

Given the importance of ensuring that responses to the youth mental health crisis are shaped by youth themselves, this review sought information about youths' requests, preferences, and demands. Youth report valuing opportunities to help others, and that being a “helper” is perceived as a more empowered state than strictly being a seeker of help—which may at times feel stigmatizing (104). In addition, youth have expressed a need for improved mental health support from schools, peers, and mental health professionals (103, 105, 106). More specifically, a survey conducted on behalf of the National Alliance on Mental Illness (NAMI) found that 66% of adolescent participants want schools to teach what mental health is, including where and how to seek treatment, and 67% would like schools to offer days off for mental health (107). Similarly, a survey conducted by Mental Health America confirmed youths' desires for mental health breaks or absences as part of school or work (103). Many youth have also cited the value of educational and non-cash resources (housing, healthcare, food) in supporting their mental wellbeing (105).

There is also a high demand among youth for public mental health awareness campaigns as well as a variety of in-person (vs. digital) resources, such as wellness centers, counselors, and therapists that offer face-to-face connection (105, 108). Youth want the ability to access mental health resources both in schools and in their communities, and they want this access to be reliable and provided by more diversity-trained, culturally competent therapists (22, 109). The desire for community-based resources and therapists apart from school may be due to concerns around confidentiality (110). Additionally, youth have reported a desire for increased mental health resources for their family members (105).

Youth have also voiced that they want more resources to support extracurricular activities outside of school (105), as these benefit their mental health. For example, youth have mentioned that listening to music daily, going to concerts or museums, playing an instrument, and engaging in sports have been key to their mental wellbeing (111–113). In fact, a study by Crisis Text Line —a non-profit that provides free text-based mental health and crisis intervention—indicated that youths’ top two support needs when in crisis were 1) opportunities for social connection, and 2) engagement in music, writing, visual, and/or performing arts (114). Elsewhere, youth have similarly voiced that arts-based methods are a means to strengthen wellbeing by offering an avenue for self-expression, building confidence, and engaging with others (115). It should be noted that out-of-school engagement with arts-based activities is impacted by socioeconomic status and resources such as transportation—indicating the need to ensure equity (113). Related to disparities in access, despite clear requests for out-of-school engagement and resources, some youth have expressed a preference for arts-based activities to be made available via school programs (109, 116–118).

Youths' interest in arts-based experiences as supports for their mental health echoes studies in which local arts and cultural activities have been associated with improved mental health, in part because they facilitate a heightened sense of community, self-expression, and cultural identity (111–113). In addition, engagement in arts activities specifically helps youth process and transform their emotions and distract from problems, sometimes in third spaces where youth can meet others with shared interests (119). These findings, in conjunction with youths' own interests and demands, underline the importance of creating inclusive, accessible, and youth-driven opportunities to engage in arts-based programming.

In addition to explicitly-requested resources, youth have described some of the barriers they experience to mental well-being and related care, which imply further needs and requests. Noted barriers include the cost of therapy, lack of insurance coverage, and transportation (108). Additional barriers include concerns about confidentiality and privacy, lack of parental understanding, social and/or internalized stigma, and lack of information about how to get help (110). Youth have also reported that therapy, longer in-depth counseling, and preventative services are less accessible than emergency services and short-term mental health counseling (108); this suggests that youth currently have more access to reactive care than to proactive, preventive resources.

This review found that, despite increasing calls to amplify youth voices in the decisions, programming, and policies that affect them (93, 94), there are still very few studies investigating what youth themselves find beneficial, and even fewer that offer open-ended questions or other forums via which youth can indicate their own preferences, needs, priorities, and proposed solutions. Moving forward, an increased effort to co-explore and co-develop strategies *with youth* will be important for effectively addressing the growing crisis (98).

### 3.3 Existing resources

The following section conveys findings related to current U.S.-based resources that are dedicated to tackling youth mental health challenges. These have been categorized as community-based, national nonprofit, or government resources.

#### 3.3.1 Community-based resources

Community-based youth mental health services play a critical role in the healthcare delivery system by reaching youths and their families in community-based settings and mitigating the need for more intense services or institutionalized care (120). In community-based settings, such as youth centers with integrated behavioral health services (121), participants may experience less cultural mistrust, racism, and discrimination in seeking mental health care compared to clinical settings and medical establishments (122). As one example, the Chicago-based Kovler Center Child Trauma Program—which provides mental health and social services to immigrant and refugee youth and families—has worked to provide culturally-relevant services and evidence-based trauma-informed therapy (123).

Community-based settings can also provide an opportunity to implement peer-led mental health interventions that may be considered more culturally acceptable (124–126). For instance, Encuentros, a strength-based, community-led support program in Maryland designed for Latino immigrant youth and families with mental health challenges, was found to increase youth participants' stress and anger management coping skills (127). Based upon these tenets of accessibility and acceptability, the WHO has called for the promotion of community mental health services over institutional care in order to deliver improved recovery outcomes for people with mental health conditions (128).

Although a comprehensive survey of available community-based resources is beyond the scope of this review, an increasing number of community arts organizations provide accessible, low- or no-cost workshops and programs for youth. For example, many museums have developed youth-focused art programs; notable examples include the American Folk Art Museum and the Museum of Contemporary Art Chicago (MCA). The American Folk Art Museum runs a program called Youth Art Connection, a free summer art program for high school students (129). The MCA offers the Teen Creative Agency, a youth leadership and creative engagement program where view art and meet with contemporary art and artists (130).

Calls for improved and expanded community-based mental health care resources are additionally grounded in the challenges noted in this review—including inadequate mental health resources, inequitable service access and engagement, and the need for improved delivery of evidence-based practices (131, 132). Notably, increased funding for community-based care would also help mitigate the effects of constant variations in nonprofit funding, which cause service disruptions and adjustments that have negative impacts on communities (133). Better funding could also reduce the difficulties that community nonprofits face in implementing federal/state regulations amidst increasing non-profit competition (134, 135).

#### 3.3.2 National nonprofit resources

National organizations and charities such as the National Alliance on Mental Illness (NAMI), Mental Health Innovations (MHI), and Mental Health America (MHA) provide support to youth through advocacy, research, education, and outreach. In keeping with findings regarding the importance of youth-led efforts, MHA has a six-month leadership program (Young Leaders Council) for people aged 18–25 in which youth are encouraged to be active agents of change by developing initiatives that meet the unique mental health needs of their communities (136). NAMI's Next GEN young adult advisory group helps inform the work of NAMI programs, content, initiatives, and new projects (137). Several other youth-led organizations promote youths' influence and engagement in mental health efforts, such as We R Native, and the chapter-based Youth Move National, which aims to improve youth-serving systems and organizations by ensuring that the voices of youth with firsthand experience are included in mental health care systems (138, 139).

More generally, many national nonprofits are focused on youth mental health specifically. The Jed Foundation, The Trevor Project, and American Foundation for Suicide Prevention (ASFP) have made it their mission to reduce youth suicide rates. For example, AFSP provides high schools around the country with the “More Than Sad” program, which teaches teens to recognize signs of depression in themselves and others, and challenges related stigma. At the time of writing, the program has reached over one million teenagers and educators (140). In addition, NAMI's “Ending the Silence,” a 50-min program that teaches adolescents to recognize the early warning signs of mental illness, has been shown to improve student knowledge and attitudes about mental health conditions, reduce negative stereotypes, and increase willingness to seek help for mental health concerns (141, 142). The #CreateConnectCare initiative centers youth voice, creative expression, and mental health, inviting youth to share their stories and build community through art, video, poetry, or movement.

Despite the aims and reach of these national programs, there remains a lack of quality studies on their effectiveness. Increased programmatic evaluation may strengthen the effectiveness, replicability, and sustainability of national youth-oriented, youth-led programs.

#### 3.3.3 Government resources

Medicaid and Children's Health Insurance Program (CHIP) serve as primary coverage sources for over 39 million youth across the nation (143). Under Medicaid, medically necessary care extends to prevention, screening, assessment, and treatment for mental health and substance use disorders (144). Connecting Kids to Coverage, a national outreach initiative that reaches out to families with youth eligible for Medicaid and CHIP, launched a campaign in May 2021 with an emphasis on mental health coverage (145). Other governmental initiatives include the 988 Suicide and Crisis Lifeline, as well as screening and training resources such as Project LAUNCH (Linking Actions for Unmet Needs in Children's Health), SAMHSA's Project AWARE (Advancing Wellness and Resiliency in Education) program, and the Infant Early Childhood Mental Health (IECMH) Grant Program.

State and federal funding has also targeted youth mental health initiatives. For example, the American Rescue Plan allocated $122 billion in relief funds to schools to support mental, emotional, social, and academic challenges faced by students (146). As noted earlier, research has indicated that youth are more likely to utilize mental health services when they are provided in school because the services take place in a familiar environment and there are fewer structural barriers including transportation (113). The Biden-Harris Administration's FY22 budget included $1.6 billion for the Community Mental Health Services Block Grant (MHBG) program (more than double the FY21 allocation) (147); which then made funds available to community mental health services. Federal funding allocation has also prioritized programs such as the Pediatric Mental Health Care Access program. This program aims to enhance access to mental health services for children and adolescents—particularly those in underserved areas—by integrating behavioral health services into pediatric primary care settings. Schools have been encouraged to use these state and federal relief funds to hire school psychologists, social workers, and counselors to serve students and their families (146). As a state-level example, Illinois launched a $10 million state grant program in 2023 to fund schools and youth healthcare agencies to strengthen the identification of mental health issues within school populations, and to provide education, resources, care coordination, and training to help improve mental health outcomes in youth (148). Further examples of federal and state funding can be found in Table 3.

**Table 3:** Examples of federal and state funding invested in targeted youth mental health initiatives

Federal	
Example	Description
American Rescue Plan	$80 million for the Pediatric Mental Health Care Access program—which aims to enhance access to mental health services for children and adolescents, particularly those in underserved areas, by integrating behavioral health services into pediatric primary care settings. One of the key approaches used is telehealth, and in 2021 approximately 8,200 children and adolescents received services through pediatric primary care providers who contacted the pediatric mental health team (143). $420 million to the Certified Community Behavioural Health Clinics (CCBHC) expansion grant program (143). $122 billion in relief funds to schools to help them support mental, emotional, social, and academic challenges faced by students (143).
Department of Health and Human Services	Nearly $35 million in funding to strengthen and expand community health services and suicide prevention programs for children and young adults (150). $190 million into providing critical community-based services such as The Children’s Mental Health Initiative, The Native Connections Grant Program, and The Youth and Family TREE Grant Program that help youth gain access to preventive treatment, early intervention, and expand on sustainable treatment plans (143). $2 million in funding per year for five years to the American Academy of Pediatrics to establish a National Center of Excellence on Social Media and Mental Wellness (150).
Office of National Drug Control Policy	$13.2 million in grants for 106 Drug-Free Communities (DFC) Support Programs that work to prevent youth substance use (143).
The White House (Biden-Harris administration)	Approximately $300 million to bolster and enhance access to mental healthcare. The Biden-Harris administration advocated for increased investment in mental health services for students (151).

State	
Example	Description
California	In 2016, California expanded the Investment in Mental Health Wellness Act to improve access to mental health crisis services for youth through allocation of funding for four areas of mental health programs, including crisis residential treatment, crisis stabilization, mobile crisis support teams, and family respite care (152).
Illinois	In November 2022, Illinois launched a $2.5 million state program to strengthen mental health services in EDs and schools. This was followed a year later by a $10 million grant program funded by the CDC’s COVID-19 Public Health Workforce Supplemental Funding program to support child and adolescent health agencies in improving student care through interventions to assist children suffering from trauma and training medical and school staff to expand adolescent mental health resources (148).
New York	In August 2023, New York State announced the availability of $108 million for school districts to create or expand programs to help students address trauma caused by the pandemic, address student learning loss, and expand school-based mental health clinics (153).
Wisconsin	In February 2022, Wisconsin launched an investment of $15 million into the “Get Kids Ahead” initiative to provide school-based mental health support and services. Funding for this program was doubled in August 2022 (154).

Despite substantial funds dedicated to addressing the youth mental health crisis, the literature indicates that youth still face multiple barriers to obtaining mental health treatment. As noted, fewer than half of U.S. adolescents have access to quality mental health care; seven states still lack the Pediatric Mental Health Care Access program; and regulatory barriers on the provision of telehealth across state lines limits the use of telehealth for youth mental health challenges (32, 73, 149). There remains a critical need to continue expanding access to healthcare, specifically mental health care, for youth across the country.

## 4 Discussion

This literature review aimed to provide an update on the current state of youth mental health in the United States, youth perspectives on mental health needs and priorities, and existing resources and gaps in addressing the youth mental health crisis. Findings reveal a concerning landscape during the critical neurodevelopmental period of adolescence, with rising rates of psychological distress among youth that are affected by a variety of factors. Notably, the review highlights significant disparities in mental health outcomes and access to care, particularly among youth from marginalized communities—underscoring the urgent need for equity-oriented, culturally responsive solutions.

A key theme emerging from this review is the critical importance of engaging youth as partners and leaders in the development and implementation of mental health solutions. The literature suggests that interventions are more effective when they incorporate youth perspectives and priorities, and when they empower youth as leaders and active agents of change. Moreover, the review indicates that youth themselves have identified a need for a comprehensive, multi-level approach that extends beyond traditional clinical settings to encompass community-based, nonclinical strategies. These include the provision of basic resources as well as initiatives that promote social connection, cultural identity, and engagement in activities such as the arts, which youth have highlighted as crucial to their mental wellbeing.

While the review identified a variety of existing resources aimed at addressing youth mental health, it also revealed ongoing challenges and gaps, such as inadequate funding, workforce shortages, inequities, and barriers to access. Addressing current challenges will require continued improvement to existing resources, including building and supporting the mental health workforce, expanding service engagement, evaluating program outcomes, and expanding healthcare enrollment and telehealth coverage for youth. More generally, effective change will require sustained investment in evidence-based, youth-led interventions that extend from clinic to community.

## 5 Limitations and future directions

While the search strategy for this literature review aimed to capture a broad range of relevant literature, the review was not designed to be as comprehensive as a scoping review; as a result, some pertinent studies may have been missed. Despite this limitation, the review offers a timely and informative synthesis of key themes and priorities in youth mental health, providing a foundation for further research and action.

Future research should build upon the findings of this review by directly engaging youth as partners and leaders in the development, implementation, and evaluation of mental health interventions. In particular, this review found gaps in data regarding youths' direct requests, priorities, and proposed solutions with regard to mental health—revealing a need for studies that elevate these inquiries. Such studies should employ culturally-responsive, participatory methods to ensure that youth voices are centered throughout both the research process and its translation into action.

## 6 Conclusion

Undertaken in the context of increasing calls to address the youth mental health crisis in the United States, this literature review further illuminates that crisis—including its current scope and contributing factors, the extent of untreated mental health conditions, and ongoing disparities in outcomes and in access to effective care. It also highlights the critical role of youth engagement and leadership in developing solutions, including the potential value of creative engagement. Though challenges persist, the review indicates that community-based resources, national organizations, and government policies and resources have the potential to adapt and respond more effectively to youth mental health concerns.

Moving forward, it is crucial to prioritize the amplification and leadership of youth voices in the co-exploration and co-development of strategies at community, state, and national levels. By seeking youth insights and leadership on needs, priorities, and decisions that affect their mental health and wellbeing, and by co-creating solutions with them to address the mental health crisis, communities and governments can make meaningful progress in providing comprehensive solutions that promote equitable wellbeing and flourishing among all youth. This approach not only addresses the immediate mental health crisis but also empowers youth as future leaders in the ongoing effort to build a more resilient, equitable, and thriving society.
